# Effect of *Limosilactobacillus* *fermentum* 332 on physicochemical characteristics, volatile flavor components, and Quorum sensing in fermented sausage

**DOI:** 10.1038/s41598-023-31161-2

**Published:** 2023-03-09

**Authors:** Yue Gu, Ruifang Qiao, Bo Jin, Yinfeng He, Jianjun Tian

**Affiliations:** College of Food Science and Engineering, Inner Mongolia Agricultural University, Hohhot, Inner Mongolia China

**Keywords:** Microbiology, Microbial communities

## Abstract

The effects of *Limosilactobacillus* *fermentum* 332 on quality characteristics in fermented sausage were explored in terms of physicochemical characteristics, volatile flavor components, and Quorum sensing (QS). The results showed that the pH of fermented sausage decreased from 5.20 to 4.54 within 24 h with the inoculation of *L.* *fermentum* 332. Lightness and redness were significantly improved, and hardness and chewiness were significantly increased after the addition of *L.* *fermentum* 332. With the inoculation of *L.* *fermentum* 332, the thiobarbituric acid reactive substance content decreased from 0.26 to 0.19 mg/100 g and total volatile basic nitrogen content decreased from 2.16 to 1.61 mg/100 g. In total, 95 and 104 types of volatile flavor components were detected in the control and fermented sausage inoculated with starter culture, respectively. The AI-2 activity of fermented sausage inoculated with *L.* *fermentum* 332 was significantly higher than that of the control and positively correlated with viable count and quality characteristics. These results provide support for further research on the effect of microorganisms on the quality of fermented food.

## Introduction

Fermented meats are produced worldwide due to their sensory characteristics and convenience^1^. A great variety of fermented meats constitutes an important part of cultural patrimony. Fermented meat products have a unique flavor and texture and long shelf life under natural or artificial conditions, which are promoted by microorganisms^2^. Meat protein is decomposed by microorganisms and enzymes to produce a large number of amino acids, which improves the nutrition and flavor of fermented meat^3^. For fermented meat, four flavor development methods are used: protein degradation, lipid oxidation, the Maillard reaction, and the use of microorganisms^4^. However, traditional natural fermentation is difficult to normalize fermented meat quality and ensure safety. Therefore, to improve product quality and reduce production difficulties by artificial inoculation of microorganisms, which can better ensure the safety and stability of fermented meat.

Lactic acid bacteria (LAB) is a microorganism that is widely used in the production of fermented meat products. LAB in fermented meats can inhibit the growth and reproduction of pathogenic and spoilage bacteria while also lowering the content of harmful substances like nitrite^5^. *Lactiplantibacillus* *plantarum* and *Streptococcus* *xylosus* in fermented sausage promoted protein and fat decomposition while inhibiting the growth of pathogenic and spoilage bacteria to prevent odor and rancidity, maintain sausage quality, and improve flavor^6^. *Lactobacillus* *sake*, for example, can promote lipid hydrolysis, inhibit lipid autoxidation, and improve fermented flavor development^7^. In terms of flavor development, LAB uses carbohydrates as the energy source to produce organic acids. Moreover, LAB can promote protein decomposition, further positively affect amino acid metabolism, and promote the development of fermented meat flavor^8^. The addition of LAB is beneficial to increasing the content of free fatty acids in fermented meat products, particularly to promote the release of unsaturated fatty acids and provide basic materials for flavor development^6,9,10^. Therefore, LAB plays an important role as a functional starter, resulting in unique flavor and safe fermented meats.

Quorum sensing (QS) is a cell density-dependent type of communication mechanism mediated by QS molecules. QS molecules, also called autoinducers (AIs), enable bacteria to collectively regulate gene expression, thereby coordinating various activities^11^. Moreover, biofilm formation of *L.* *sanfranciscensis*^12^, cell adhesion of *L.* *acidophilus*^13^, and environmental tolerance of gram-positive bacteria^14^ can be controlled by QS systems. AIs are classified into several types, with AI-2 being the only signal molecule produced by both gram-negative and gram-positive bacteria and used for intraspecific or interspecific communication^15^. AI-2 is produced by S-ribosylhomocysteinase (LuxS), an enzyme found in many bacterial species that has been proposed to allow interspecies communication. The *luxS* gene has been identified in food-borne LAB, including *Lactobacillus* spp.^16^ and *Bacillus* spp.^17^, as well as food-borne pathogens^18^. Our previous research has shown that the LuxS/AI-2 QS system can regulate LAB metabolism and influence physiological activities^19,20^. Therefore, AI-2 might affect the quality of fermented food by regulating the metabolism of LAB. However, the QS and AI of LAB are seldom investigated in fermented foods. The knowledge of the QS of LAB during meat fermentation is rather preliminary and based on empirical observations. The relationship between AI-2 and the quality characteristics of fermented sausage has not been reported previously.

*Limosilactobacillus* *fermentum* 332, previously isolated from fermented food with good fermentation potential for meat products and high AI-2 activity, was used as the starter for fermented sausage in this study. The changes in the activity of the signal molecule AI-2 and the quality of fermented sausage during the manufacturing process were investigated. The potential relationship between LAB AI-2 and flavors in fermented sausage was investigated preliminary, laying the theoretical groundwork for the directional improvement of fermentation strain production performance and product quality in the food industry.

## Results and discussion

### LAB viable count and pH changes

**Figure 1:**
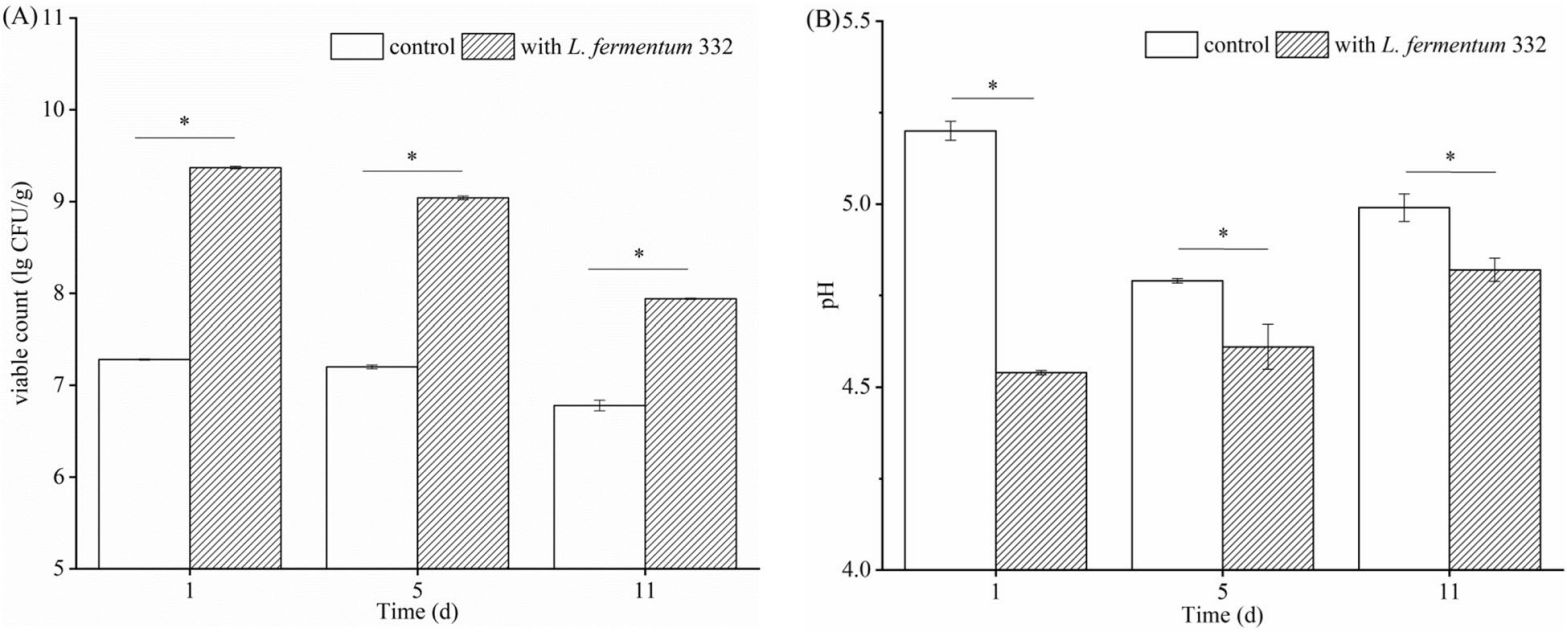
Changes of *L.* *fermentum* 332 viable count (**A**) and pH (**B**) of fermented sausage.*Indicate significant difference (*p* < 0.05).

The change in LAB viable count is shown in Fig. 1A. The LAB viable count of fermented sausage, inoculated with *L.* *fermentum* 332, was significantly higher than that in control on days 1, 5, and 11 (*p* < 0.05). The LAB viable count was highest during the fermentation (day 1) period, then decreased during the drying (day 5) and mature periods (day 11). As shown in Fig. 1B, the pH of the fermented sausage inoculated with starter culture decreased rapidly from 5.64 to 4.54 within 24 h, while the pH of the control decreased from 5.65 to 5.20. The pH of the inoculated group increased slightly after 24 h (1 day) of fermentation. The pH rose to 4.82 on day 11. The fermented sausage itself contained some LAB and caused the pH decrease in fermented sausage without *L.* *fermentum* 332 (control). The pH of control was the lowest on day 5, then slightly increased to 4.99 on day 11. During the initial stage of fermentation, optimal temperature and humidity were required for the growth of microorganisms. *L.* *fermentum* 332 was grown and metabolized rapidly under optimal conditions, producing a large amount of lactic acid and other acids. Therefore, the pH decreased rapidly, and the acidity increased. After, the microorganisms in the meat interact with the enzymes to further decompose the proteins to produce free amino acids, ammonia, and basic amines, which slightly increase the pH value^21,22^. The pH of the inoculated group was significantly lower than that of the control during the 1–11 days of fermentation (*p* < 0.05). These results were consistent with the results of other studies that reported the pH of the fermented sausage inoculated with LAB was lower than the pH of the control sausage^23–28^. The result showed that *L.* *fermentum* 332 has strong acidifying property, which effectively decreases the fermentation time. Meanwhile, whether the acidity change of fermented sausage caused by inoculation of *L.* *fermentum* 332 was related to the QS needed further study.

### Color changes

Sausage color is crucial for the marketability of the meat product because it influences its appearance and acceptability. The color indexes include lightness (L*), redness (a*), and yellowness (b*). L* indicates the brightness of the sausage. The higher L* value indicates a brighter color of the sausage. a* indicates the bright red degree of sausage. The sausage products show attractive rose colors when the L* and a* of sausage are higher. The color changes of fermented sausage are presented in Table 1. The L* of the two groups of fermented sausage showed a downward trend. On days 1 and 5, the L* of the fermented sausage inoculated with starter culture was significantly higher than that of the control (*p* < 0.05). On day 11, the L* of the fermented sausage inoculated with starter culture was lower than that of the control (*p* < 0.05). A similar result was reported^29^. The addition of *L.* *fermentum* 332 reduced the *a*_*w*_ of the fermented sausage and dried the surface of the fermented sausage. a* of fermented sausage showed an upward trend. During the mature stage (day 11), a* value of the fermented sausage inoculated with starter culture was significantly higher than that of the control (*p* < 0.05). No significant difference was found in b* between the two groups. b* is an important variable related to the lipid^29^. The results indicated that the addition of *L.* *fermentum* 332 was beneficial in improving the color of the sausage. Previous studies pointed that the LAB had the effect of improving color in fermented sausages^30,31^.

**Table 1:** Effect of *L.* *fermentum* 332 on fermented sausage color.

Color	Time (days)	Control	With *L.* *fermentum* 332
L*	1	44.53 ± 0.66^b^	46.8 ± 0.11^a^
	5	43.82 ± 0.09^b^	45.59 ± 0.44^a^
	11	41.67 ± 0.14^a^	40.59 ± 0.38^b^
a*	1	16.67 ± 0.32^a^	16.7 ± 0.19^a^
	5	16.83 ± 0.44^a^	16.87 ± 0.33^a^
	11	16.99 ± 0.14^b^	17.50 ± 0.10^a^
b*	1	9.71 ± 0.50^a^	9.23 ± 0.15^a^
	5	9.34 ± 0.31^a^	9.04 ± 0.27^a^
	11	7.94 ± 0.04^a^	7.82 ± 0.11^a^

The color values were expressed as mean ± standard deviation (n = 3). Different letters at the same time indicate significant difference (*p* < 0.05).

### Texture changes

Texture indices included hardness, elasticity, adhesiveness, and chewiness, all of which were affected by the meat's ripening time. For control and inoculated sausages, hardness, adhesiveness, and chewiness increased significantly with ripening time (Table 2). The increase in hardness caused by sausage ripening was primarily due to water loss. These results were consistent with others^32,33^. Instead, elasticity decreased through ripening time. The results of instrumental texture showed significant differences between the control and inoculated sausages. The addition of *L.* *fermentum* 332 significantly increased the hardness, adhesiveness, and chewiness and decreased the elasticity (*p* < 0.05). This may be because *L.* *fermentum* 332 increased the degradation of proteins. The reduction of sulfhydryl content caused an increase in hardness^34^. The result indicated that the addition of *L.* *fermentum* 332 changed the texture characteristics of the fermented sausage.

**Table 2:** Effect of *L.* *fermentum* 332 on fermented sausage texture.

		1 day	5 days	11 days
Hardness (g)	Control	456.09 ± 58.21^b^	1293.86 ± 86.31^b^	1792.71 ± 126.5^b^
	*L.* *fermentum* 332	835.68 ± 26.07^a^	2184.11 ± 245.5^a^	4142.37 ± 28.21^a^
Adhesiveness (mm)	Control	0.64 ± 0.04^b^	0.66 ± 0.03^a^	0.57 ± 0.05^a^
	*L.* *fermentum* 332	0.68 ± 0.05^a^	0.58 ± 0.02^b^	0.51 ± 0.01^b^
Chewiness (g)	Control	143.92 ± 36.24^b^	574.54 ± 37.83^b^	661.66 ± 40.98^b^
	*L.* *fermentum* 332	321.31 ± 25.25^a^	679.25 ± 50.7^a^	925.9 ± 94.66^a^

The texture indexes were expressed as mean ± standard deviation (n = 3). Different letters at the same time indicate significant difference (*p* < 0.05).

### TBARS and TVBN changes

**Figure 2:**
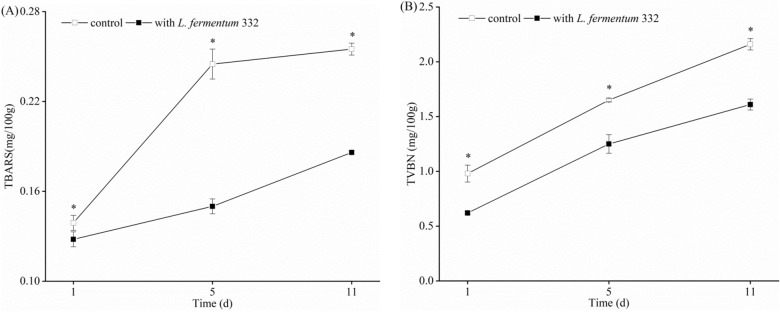
The impact of *L.* *fermentum* 332 on TBARS (**A**) and TVBN (**B**). *Indicate significant difference (*p* < 0.05).

The TBARS value is the most commonly used to assess lipid oxidation in meat products. Carbonyls, aldehydes, and hydrocarbons are the main TBARS components that contribute to off-aromas and flavors in meat products. The TBARS of the two fermented sausage groups increased, as shown in Fig. 2A. The TBARS value of control and inoculated sausages gradually increased. The TBARS value of the fermented sausage inoculated with starter culture was significantly lower than that of the control from day 1 to 11 (*p* < 0.05). With the addition of *L.* *fermentum* 332, the TBARS decreased from 0.255 to 0.186 mg/100 g on day 11. It has been reported that the TBARS values were between 0.40 and 3.90 mg MDA/kg for vacuum-packed sausages^35^. The organic sausages did not exceed the value of 3.0 mg/kg, which was used as an indicator of meat oxidative rancidity^36^. In contrast to our findings, adding acid whey and probiotic strains to the experimental model fermented sausage had no effect (*p* > 0.05) on TBARS values after 0, 90, and 180 days of storage when compared to the organic sample with sea salt^36^. The disparity is most likely due to the different antioxidant activities of different strains.

TVBN is the major product of protein decomposed by bacteria in meat, in which protein provides rich nutrition for microorganism growth. The effect of *L.* *fermentum* 332 addition on TVBN in fermented sausage was shown in Fig. 2B. The TVBN content of the two fermented sausage groups increased as time passed. The control's TVBN content ranged from 0.98 to 2.16 mg/100 g. The TVBN content of fermented sausage inoculated with starter culture ranged between 0.62 and 1.61 mg/100 g. From day 1 to day 11, the TVBN content of the fermented sausage inoculated with starter culture was significantly lower than that of the control (*p* < 0.05). Therefore, the *L.* *fermentum* 332 inoculation delayed the increase of TVBN and improved the quality of the fermented sausage.

### Volatile flavor components changes

The main sources of volatile flavor components are lipid oxidative decomposition, protein degradation metabolism, and carbohydrate decomposition^37^. Aldehydes, ketones, esters, acids, alcohols, and other substances would be produced during the fermentation of sausage. The quality of fermented sausage is affected by various components. The effect of adding *L.* *fermentum* 332 on volatile flavor components in fermented sausage is shown in Table 3. In the two fermented sausage groups, 121 volatile flavor compounds were detected, including 27 alcohols, 11 aldehydes, 37 esters, 6 ketones, 14 acids, 14 alkenes, 4 alkanes, 2 phenols, and 6 benzenes. The majority of the compounds found in fermented sausages have previously been reported^38,39^. These compounds are typically formed as a result of protein and lipid oxidation, amino acid metabolism, and carbohydrate catabolism^40^. There were 95 different types of volatile flavor substances detected in the control, and 104 different types of volatile flavor substances were detected in the fermented sausage inoculated with starter culture. The proportion of esters and alcohol in the two groups' sausages was significantly higher than the proportion of other volatile flavor components.

**Table 3:** Volatile flavor components identified and quantified by gas chromatography/mass spectrometry in fermented sausages during fermentation.

Volatile flavor components	Quantity						Content					
	1 day		5 days		11 days		1 day		5 days		11 days	
	Control	Treatment	Control	Treatment	Control	Treatment	Control	Treatment	Control	Treatment	Control	Treatment
Alcohols	13	18	11	16	12	13	360.7 ± 11.62^Ba^	384.73 ± 18.09^Aa^	235.17 ± 16.6^Bc^	336.51 ± 18.75^Ab^	280.7 ± 16.3^Ab^	257.16 ± 9.60^Bc^
Aldehydes	4	5	6	4	7	7	46.81 ± 3.83^Bc^	87.07 ± 5.91^Ab^	224.5 ± 34.36^Ab^	57.3 ± 1.28^Bc^	232.7 ± 5.55^Aa^	94.21 ± 8.06^Ba^
Esters	17	18	15	23	23	25	167.3 ± 12.15^Bc^	307.49 ± 25.71^Ac^	304.33 ± 28.05^Bb^	458.85 ± 45.537^Aa^	339.9 ± 30.64^Ba^	402.34 ± 23.96^Aa^
Ketones	5	5	4	4	4	4	27.38 ± 2.95^Aa^	16.05 ± 1.25^Ba^	12.58 ± 1.78^Bb^	13.44 ± 1.03^Ab^	12.8 ± 0.91^Bb^	13.46 ± 0.54^Ab^
Acids	5	4	7	7	6	7	29.72 ± 2.92^Bc^	77.73 ± 3.51^Ab^	51.24 ± 9.05^Bb^	116.87 ± 6.34^Aa^	103.05 ± 6.56^Aa^	42.79 ± 3.99^Bc^
Alkenes	11	9	6	7	7	8	40.24 ± 2.82^Aa^	39 ± 1.96^Ab^	29.44 ± 3.76^Bb^	41.45 ± 2.34^Aa^	29.46 ± 2.21^Bb^	38.89 ± 1.91^Ab^
Alkanes	4	2	3	2	3	3	3.08 ± 0.32^Ab^	3.07 ± 0.37^Ab^	7.43 ± 0.72^Aa^	2.78 ± 0.14^Bc^	7.13 ± 0.97^Aa^	6.55 ± 0.33^Ba^
Phenols	0	1	0	1	1	2	–	9.33 ± 0.19^Aa^	–	3.21 ± 0.30^Ac^	6.56 ± 0.42^Aa^	5.79 ± 0.17^Bb^
Benzene	3	3	2	2	4	4	11.84 ± 0.77^Ba^	16.56 ± 1.14^Aa^	6.52 ± 0.083^Ab^	6.06 ± 0.69^Ac^	11.47 ± 0.22^Aa^	9.91 ± 0.30^Bb^

Data are presented as mean ± standard deviation. Capital letters represent the significant difference between different groups at the same stage on the same line (*p* < 0.05). Lowercase letters represent the significant difference between different stages in the same group on the same line (*p* < 0.05).– indicate not checked out.

On day 1 (fermentation stage), 62 different volatile flavor compounds were detected in the control culture and 65 different volatile flavor compounds in the fermented sausage inoculated with starter culture. Alcohols and esters had significantly higher types and contents than other types (*p* < 0.05), while aldehydes, ketones, acids, olefins, and other types had relatively lower contents. Because of the addition of *L.* *fermentum* 332, the content of alcohol and ester in the fermented sausage inoculated with starter culture was significantly higher than in the control (*p* < 0.05). Esters are created by esterifying alcohols and acids. The majority of them have fruit and flower fragrances and contribute significantly to the flavor formation of meat products. As shown in Supplementary Tables S1 and S2, eucalyptol, ethyl caproate and ethyl octanoate showed higher content than others in fermented sausage inoculated with starter culture and control. The addition of *L.* *fermentum* 332 increased the content of ethyl caproate and ethyl octanoate in fermented sausage, and both were significantly higher than the control (*p* < 0.05). The alcohol types in the fermented sausage inoculated with starter culture were significantly higher than those in the control. Aldehydes are flavor compounds found in fermented sausage. *N*-hexanal, the basic product of linoleic acid oxidation, was found in the fermented sausage inoculated with starter culture. It has a grassy odor and reflects the degree of fat oxidation, but it was not detected in the control. Therefore, the addition of *L.* *fermentum* 332 increased the amount of flavor substances in the fermentation stage.

On day 5 (drying stage), 54 types of volatile flavor compounds were detected in the control and 66 types of volatile flavor compounds in the fermented sausage inoculated with starter culture. The variety of volatile flavor compounds was lower in the drying stage than in the fermentation stage, which could be attributed to environmental changes.

On day 11 (mature stage), a total of 67 types of volatile flavor compounds were detected in the control and 73 types of volatile flavor compounds in the fermented sausage inoculated with starter culture. The types and concentrations of aldehydes in the fermented sausage inoculated with starter culture were significantly higher than those in the other processing stages (*p* < 0.05), indicating a higher degree of lipid oxidation. Esters are volatile compounds that contribute to the distinct flavor of fermented meat^41^. Esters accounted for 21.31% of the total volatile substances in the fermented sausage inoculated with starter culture, and the number of species was 25. The proportion of esters in total volatile substances was 18.1% in the control, and the number of species was 23. The types and contents of olefins in the fermented sausage inoculated with starter culture were greater than in the control culture (*p* < 0.05). Therefore, adding *L.* *fermentum* 332 improved the flavor of the fermented sausage.

### ***a***_***w***_ changes

**Figure 3:**
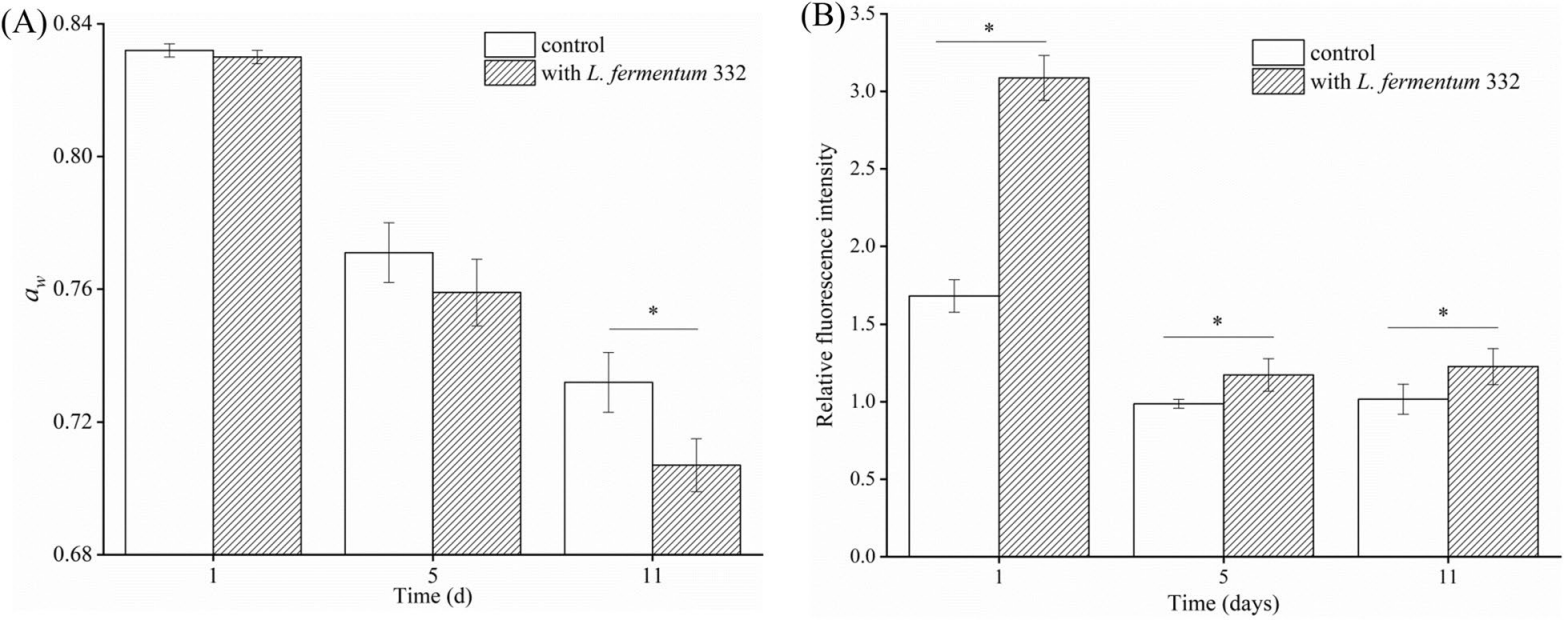
The impact of *L.* *fermentum* 332 on *a*_*w*_ (**A**) and AI-2 activity (**B**).*Indicate significant difference (*p* < 0.05).

As shown in Fig. 3A, the addition of *L.* *fermentum* 332 to the fermented sausage significantly decreased *a*_*w*_ compared with that of the control (*p* < 0.05). *a*_*w*_ of the inoculated group decreased from 0.830 to 0.707, and that of the control decreased from 0.832 to 0.732 after 11 days of fermentation. The differences were due to the evaporation of the surface moisture and water migration inside the sausages during the fermentation^42^. Our results were consistent with other reported results^32^. Therefore, the addition of *L.* *fermentum* 332 can effectively reduce the *a*_*w*_ of fermented sausage, thereby extending the shelf life of the product.

### AI-2 activity changes

The change of AI-2 activity during the fermentation of sausage is shown in Fig. 3B. The AI-2 activity was detected in the fermented sausage samples. It has been reported that the AI-2 activity detected in different types of Kimchi was different, which was related to the different LAB strains in Kimchi^43^. The AI-2 activity of LAB strains, which isolated from fermented meat was detected^11^. Moreover, the addition of nitrate increased the *luxS* gene expression. The AI-2 activity of fermented sausage, which inoculated *L.* *fermentum* 332, was significantly higher than that in the control on days 1, 5, and 11 (*p* < 0.05). The AI-2 activity was highest in the fermentation period (day 1), then decreased during the drying period (day 5) and aging period (day 11). The change in AI-2 activity was similar to the change in LAB viable count. This indicated that the activity of AI-2 was higher in the fermented sausage inoculated with starter culture fermented sausage and consistent with the viable count of the strain. QS not only related to bacterial density but also affected by the surrounding media^44^.

The results showed the AI-2 activity and acid production of fermented sausage inoculated with starter culture were both significantly higher than those of the control (*p* < 0.05). This may be because the higher LAB viable count number caused the increase in AI-2 activity, which accelerated the acid production *L.* *fermentum* 332.

In the fermentation stage, the brightness and redness of the starter control were significantly higher than that of the control, according to the analysis of the potential correlation between AI-2 activity and color changes (*p* < 0.05). The addition of *L.* *fermentum* 332 improved the color of the sausage over the control. This may be because *L.* *fermentum* 332 decreased the pH, and promoted nitrite to combine with myoglobin to form nitromyoglobin. Meanwhile, the hardness and chewiness of fermented sausage inoculated with starter culture were significantly higher than those of the control (*p* < 0.05). At this point, the fermented sausage inoculated with starter culture displayed increased AI-2 activity, which is involved in many physiological metabolic processes of LAB^19,20^. The increased AI-2 activity might promote the metabolism of *L.* *fermentum* 332, influencing the color and texture of fermented sausage.

The analysis of potential correlations between AI-2 activity, TBARS, and TVBN changes revealed that AI-2 activity was negatively correlated with TBARS and TVBN values. The AI-2 activity of the fermented sausage inoculated with starter culture was significantly higher than the control, but the TBARS and TVBN values were significantly lower. It was suggested that there might be potential correlation among AI-2 activity, lipid oxidation and protein decomposition. The exact relationship needs to be further studied.

The potential correlation between AI-2 activity and volatile flavor component changes was investigated, and it was discovered that the types of volatile flavor substances in the fermented sausage inoculated with starter culture (where AI-2 activity was higher) were significantly higher than those in the control (*p* < 0.05). This showed that adding *L.* *fermentum* 332 increased the types of volatile flavor components. Therefore, AI-2 might take part in the formation of flavor substances. However, the distinct relationship between the volatile flavor components and AI-2 activity warrants further investigation.

In conclusion, with the inoculation of *L.* *fermentum* 332 the quality of fermented sausage was improved. However, there has yet to be published research on the relationship between AI-2 and the quality characteristics of fermented sausage. The potential relationship between AI-2 and the quality characteristics of the fermented sausage was investigated in this study. AI-2 activity of fermented sausage increased with the inoculation of *L.* *fermentum* 332, which was accompanied by a decrease in pH, an improvement in color and texture, a decrease in TBARS and TVBN values, and an increase in volatile flavor substances. These changes, in general, have an impact on the development of flavor compounds in fermented sausage. Therefore, AI-2 activity might influence the quality characteristics of fermented sausage. More research is needed to determine the mechanism underlying the effect of AI-2 activity on the quality characteristics of fermented sausage during fermentation.

## Materials and methods

### Strains and growth conditions

*Limosilactobacillus* *fermentum* 332 was isolated from Chinese traditional fermented foods and kept in MRS broth supplemented with 20% (v/v) glycerol as frozen (80 °C) stocks. Before use, it was transferred at least three times in MRS broth (Solarbio, Beijing, China) at 37 °C. *Vibrio* *harveyi* BB170 is a directionally mutated strain with an AI-2 receptor that can be used to measure AI-2 activity^45^. *V.* *harveyi* BB170 (ATCC BAA-1117) was cultured at 30 °C with shaking after being transferred at least three times in an autoinducer bioassay (AB) medium (Huankai, Guangdong, China)^46^.

### Preparation of fermented sausages

Fresh mutton hindleg meat and tail fat were obtained from a local commercial processor (Hohhot, China), and sausages were made with modifications to the method previously described^47^. The sausage's ingredients were as follows: mutton hindleg meat (70%), mutton tail fat (30%), salt (2.5%), glucose (0.5%), sugar (1%), NaNO_2_ (0.01%), ascorbic acid (0.05%), pepper powder (0.2%), ginger powder (0.2%), spice powder (0.1%), corn starch (1%), and lactalbumin powder (0.5%). The ingredients were thoroughly mixed and filled into pig casings. The sausage diameter was approximately 3 cm, and length was approximately 20 cm. Control was fermented sausage without starter culture and the treatment was fermented sausage inoculated with *L.* *fermentum* 332 (the concentration of the starter culture was 4%, 10^6^ CFU/g). For 24 h, fermentation was carried out at 30 °C and 95% relative humidity (day 1). This was known as the fermentation period. For four days, the sausages were placed in a 15 °C and 75–85% relative humidity environment (day 5). This stage was regarded as the drying period. Then, the sausages were transferred to an environment of 10 °C and 65% relative humidity for 6 days (day 11). This stage was regarded as the maturation period. After preparation, the samples were packed and stored at − 20 °C until further analyses. The sausages in both groups were sampled at various fermentation times (day 1, 5, and 11) to determine AI-2 activity, LAB viable count, physicochemical characteristics, and volatile flavor components.

### LAB viable count of fermented sausages

Plate counts were used to determine LAB viable counts according to the method previously described^48^.

### Physicochemical characteristics of fermented sausages

#### pH

The sausage samples were homogenized with 10 times the mass of potassium chloride solution, and the filtrate was collected to measure the pH value using a PB-10 pH meter (Sigma-Aldrich, St. Louis, USA).

#### Color

The sausage color was assessed using a TCP2 chromometer (Nanjing Bei Instrument Equipment Co., Ltd, Jiangsu, China). The lightness (L*), redness (a*), and yellowness (b*) values of each sample were measured.

#### Texture

The sausage sample was cut into 1 × 1 × 1 cm^3^, and the texture was assessed using a QTS texture analyzer (Food Technology Corporation, Los Angeles, USA). The hardness (g), elasticity (mm), and chewiness (g) values of each sample were measured.

### Thiobarbituric acid reactive substance (TBARS)

To determine the degree of lipid oxidation, the TBARS of sausage samples was quantified. Shaking for 30 min, a 10-g minced sausage sample was mixed with 50 mL of 7.5% trichloroacetic acid (containing 0.1% ethylenediaminetetraacetic acid). Following that, 5 mL of the supernatant was filtered and mixed with 5 mL of 0.02 mol/L thiobarbituric acid solution at 90 °C for 40 min. 5 mL of chloroform was added after the mixed solution had cooled. A multifunctional microplate reader was used to measure absorbance at 532 and 600 nm (BioTek Epoch, Vermont, USA). The following equation was used to calculate the TBARS value:

$$\mathrm{TBARS }(\mathrm{mg}/100\mathrm{ g})=\frac{\mathrm{A}532-\mathrm{A}600}{155}\times \left(\frac{1}{10}\right)\times 72.6 \times 100.$$

Here, A532 and A600 are the absorbances (532 and 600 nm) of the assay solution.

### Total volatile basic nitrogen (TVBN)

The TVBN content was determined using the method previously described^49^ with slight modifications. Of note, 5 g of the sausage sample was blended with 25 mL of distilled water and equilibrated for 30 min at room temperature. Filter paper was used to filter the solution. By adding 5 mL of 10 g/L magnesia, a 10-mL filtrate was made alkaline and distilled for 5 min. A control of 10 mL of distilled water was also used. The distillate was collected in an Erlenmeyer flask with 10 mL of 20 g/L boric acid aqueous solutions and a mixed indicator made by dissolving 0.1 g of methyl red and 0.5 g of bromocresol green into 100 mL of 95% ethanol. Titration with 0.01 mol/L hydrochloric acid solution was performed on the mixed solution. The TVBN content was calculated using the following equation:

$$\mathrm{TVBN}\left(\mathrm{mg}/100\mathrm{ g}\right)=\frac{\left[\left(\mathrm{V}1-\mathrm{V}2\right)\times \mathrm{c}\times 14\right]}{\mathrm{m}\times \frac{10}{100}}\times 100.$$

Here, V1 is the titration volume of the tested sample (mL), V2 is the titration volume of the blank (mL), c is the actual concentration of hydrochloric acid (mol/L), and m is the weight of the sausage sample (g).

### Water activity

Water activity (*a*_*w*_) was measured using an HD-3A water activity meter (Wuxi Huake Instrument Co., Ltd, Jiangsu, China).

### Volatile flavor components

Volatile flavor components were assessed using the method previously described^50^. The solid phase microextraction technique was used to extract the headspace volatile compounds. Of note, 5 g of the sausage sample was minced. Each sample was exposed to a solid phase microextraction fiber (DVB/CAR/PDMS 50/30 m; 57328-U; Supelco, Bellefonte, PA, USA), and extraction was performed for 40 min at 60 °C. After extraction, the fiber was inserted into the injection port and desorbed for 3 min at 250 °C. A gas chromatography/mass spectrometry system was used to analyze volatile compounds (TRACE 1300; Thermo Fisher Scientific, Waltham, MA, USA). The protocol was carried out exactly as previously described^51^. As an internal standard, 2-methyl-3-heptanone was used. Volatile compounds were identified using mass spectra obtained from the NIST MS Search 2.0 library database.

### AI-2 activity of fermented sausages

Minced fermented sausage and sterile distilled water were mixed at a ratio of 1:1 (w/v). The supernatant was collected after centrifugation at 12,000×*g* for 10 min, and the pH value was adjusted to 7.0. Next, the supernatant was filtered using a bacterial filter (0.22 µm; Linghang Technology Co., Ltd, Tianjin, China) for sterilization. The supernatant was stored at − 80 °C until further analyses. AI-2 activity was evaluated using *V.* *harveyi* BB170 as described previously^52^. *V.* *harveyi* BB170 was grown in AB medium at 30 °C with shaking. The resulting cells were diluted in fresh AB medium (5000-fold dilution; approximately 10^5^ CFU/mL) to OD_595 nm_ = 0.7–1.2. Diluted *V.* *harveyi* BB170 was mixed with fermented sausage supernatant in a 100:1 (v/v) ratio. The mixture was shaken and cultured at 30 °C. The luminescence of the samples was quantified using a VICTOR X Light Luminescence Plate Reader (Perkin Elmer, Waltham, USA).

### Statistical analysis

All tests were repeated at least three times. Results are expressed as the mean ± standard error. Data analysis was performed using SPSS 1.0 software (IBM Corporation, Armonk, NY, USA). A t-test was used to compare significant differences (*p* < 0.05) between the two groups of fermented sausages.

## Supplementary Information

Supplementary Table S1.

Supplementary Table S2.

## Data Availability

The datasets used and/or analyzed during the current study available from the corresponding author on reasonable request.
