# Supplementary material for: Effect of Limosilactobacillus fermentum 332 on physicochemical characteristics, volatile flavor components, and Quorum sensing in fermented sausage
Source: Sci Rep. 2023 Mar 9;13:3942. doi: 10.1038/s41598-023-31161-2 (PMC9998864; doi:10.1038/s41598-023-31161-2)
Supplement: Supplementary file 1 — Supplementary Table S1. [file 41598_2023_31161_MOESM1_ESM.docx]

**Table S1** The volatile flavor compounds of fermented sausage inoculated with starter culture

| Volatile compounds |  | Name | Molecular formula | 1 d | 5 d | 11 d |
| --- | --- | --- | --- | --- | --- | --- |
| Alcohols | 1 | Ethanol | C_2_H_6O_ | 46.01±8.25^a^ | 0.51±0.06^b^ | 0.29±0.04^bc^ |
|  | 2 | 2,3-Butanediol, [S-(R*,R*)]- | C_4_H_10_O_2_ | 44.15±3.05^a^ | 45.35±2.4^a^ | - |
|  | 3 | 2,3-Butanediol | C_4_H_10_O_2_ | 4.53±0.46^b^ | - | 9.09±0.5^a^ |
|  | 4 | 1-Hexanol | C_6_H_14_O | 5.42±0.44^a^ | 3.29±0.72^b^ | - |
|  | 5 | 2-Hexanol, 3-methyl- | C_7_H_16_O | 1.92±0.02^a^ | 1.99±0.03^a^ | - |
|  | 6 | 1-Heptanol | C_7_H_16_O | 3.95±0.18^a^ | - | - |
|  | 7 | Eucalyptol | C_10_H_18_O | 165.44±1.22^a^ | 142.93±8.91^b^ | 110.48±2.47^c^ |
|  | 8 | 1-Octanol | C_8_H_18_O | 9.94±0.48^a^ | - | - |
|  | 9 | 1,6-Octadien-3-ol, 3,7-dimethyl- | C_10_H_18_O | 37.16±2.2^b^ | 40.23±1.1^a^ | 32.24±1.51^c^ |
|  | 10 | endo-Borneol | C_10_H_18_O | 12.04±0.43^a^ | 10.67±0.6^b^ | - |
|  | 11 | 1-Nonanol | C_9_H_20_O | 8.61±0.23^a^ | 6.39±0.696^b^ | 4.58±0.43^c^ |
|  | 12 | Terpinen-4-ol | C_10_H_18_O | 6.05±0.23^a^ | 4.08±0.12^b^ | 3.997±0.3^b^ |
|  | 13 | α-Terpineol | C_10_H_18_O | 29.79±0.05^a^ | 27.24±2.32^ab^ | 25.27±1.65^bc^ |
|  | 14 | 2,6-Octadien-1-ol, 3,7-dimethyl-, (Z)- | C_10_H_18_O | 3.62±0.44^a^ | - | - |
|  | 15 | 1,6,10-Dodecatrien-3-ol, 3,7,11-trimethyl-, [S-(Z)]- | C_15_H_26_O | 0.5±0.02^a^ | - | 0.72±0.006^a^ |
|  | 16 | 2-Hexadecanol | C_16_H_34_O | 1.01±0.11^a^ | 0.72±0.01^b^ | 0.73±0.07^b^ |
|  | 17 | Bicyclo[3.1.1]hept-2-ene-2-ethanol, 6,6-dimethyl- | C_11_H_18_O | 4.59±0.28^a^ | - | - |
|  | 18 | 2,3-Butanediol, [R-(R*,R*)]- | C_4_H_10_O_2_ | - | 40.93±1.15^b^ | 63.32±2.37^a^ |
|  | 19 | 1-Octen-3-ol | C_8_H_16_O | - | 3.02±0.45^a^ | - |
|  | 20 | Geraniol | C10H18O | - | 4.43±0.06^a^ | 4.5±0.23^a^ |
|  | 21 | 1-Dodecanol | C_12_H_26_O | - | 3.83±0.07^a^ | - |
|  | 22 | 2-Heptanol | C_7_H_16_O | - | - | 1.59±0.01^a^ |
|  | 23 | Diethylene glycol monododecyl ether | C_16_H_34_O_3_ | - | - | 0.36±0.01^a^ |
| Aldehyde | 24 | Heptanal | C_7_H_14_O | 9.81±1.38^a^ | 5.23±0.59^c^ | 8.91±0.27^ab^ |
|  | 25 | Nonanal | C_9_H_18_O | 71.67±3.93^a^ | 47.13±0.42^b^ | 70.5±6.1^a^ |
|  | 26 | 2-Undecenal, E- | C_11_H_20_O | 1.05±0.1^a^ | 0.89±0.15^b^ | 1.29±0.06^a^ |
|  | 27 | Decanal | C_10_H_20_O | 3.33±0.24 | 4.05±0.12^a^ | 3.76±0.1^a^ |
|  | 28 | N-hexanal | C_6_H_12_O | 1.21±0.26^a^ | - | - |
|  | 29 | Methional | C_4_H_8_OS | - | - | 0.53±0.09^a^ |
|  | 30 | 2(3H)-Furanone, 5-heptyldihydro- | C_11_H_20_O_2_ | - | - | 0.76±0.08^a^ |
|  | 31 | Benzaldehyde | C_7_H_6_O | - | - | 8.46±1.36^a^ |
| Esters | 32 | Butanoic acid, ethyl ester | C_6_H_12_O_2_ | 28.15±0.6^a^ | 28.13±3.94^a^ | 20.74±0.62^b^ |
|  | 33 | Propanoic acid, 2-hydroxy-, butyl ester | C_7_H_14_O_3_ | 21.97±2.17^a^ | - | - |
|  | 34 | Butanoic acid, 3-methyl-, ethyl ester | C_7_H_14_O_2_ | 4.98±0.54^bc^ | 8.43±0.75^a^ | 6.23±0.37^b^ |
|  | 35 | Pentanoic acid, ethyl ester | C_7_H_14_O_2_ | 2.5±0.26^bc^ | 5.84±0.38^a^ | 3.64±0.05^b^ |
|  | 36 | Ethyl caproate | C_8_H_16_O_2_ | 124.18±9.86^c^ | 165.95±26.1^a^ | 153.41±6.22^b^ |
|  | 37 | Benzoic acid, ethyl ester | C_9_H_10_O_2_ | 1.65±0.21 | 2.47±0.017^a^ | 2.46±0.159^a^ |
|  | 38 | Ethyl octanoate | C_10_H_20_O_2_ | 70.76±7.42^c^ | 74.12±2.26^b^ | 78.56±4.27^a^ |
|  | 39 | 1,6-Octadien-3-ol, 3,7-dimethyl-, 2-aminobenzoate | C_17_H_23_NO_2_ | 5.36±0.35^a^ | 4.96±0.26^b^ | 5.28±0.371^a^ |
|  | 40 | Nonanoic acid, ethyl ester | C_11_H_22_O_2_ | 3.37±0.29^a^ | - | 4.05±0.28^a^ |
|  | 41 | Geranyl isovalerate | C_15_H_26_O_2_ | 1.04±0.19^a^ | - | - |
|  | 42 | 3-Cyclohexene-1-methanol, α,α,4-trimethyl-, acetate | C_12_H_20_O_2_ | 8.05±0.27^a^ | 5.59±0.15^bc^ | 6.35±0.944^b^ |
|  | 43 | 1,6-Octadien-3-ol, 3,7-dimethyl-, formate | C_11_H_18_O_2_ | 0.51±0.06^a^ | - | - |
|  | 44 | Decanoic acid, ethyl ester | C_12_H_24_O_2_ | 22.81±1.71^c^ | 30.6±4.4^b^ | 38.31±1.799^a^ |
|  | 45 | 5,8,11,14-Eicosatetraenoic acid, methyl ester, (all-Z)- | C_21_H_34_O_2_ | 0.5±0.01^b^ | 0.48±0.05^b^ | 1.36±0.038^a^ |
|  | 46 | Dodecanoic acid, ethyl ester | C_14_H_28_O_2_ | 1.7±0.23^b^ | 1.98±0.27^b^ | 2.67±0.091^a^ |
|  | 47 | Hexadecanoic acid, ethyl ester | C_18_H_36_O_2_ | 0.7±0.07^b^ | 0.79±0.08^b^ | 1.16±0.036^a^ |
|  | 48 | 1-Butanol, 3-methyl-, formate | C_6_H_12_O_2_ | 8.68±1.39^a^ | - | 6.49±0.21^b^ |
|  | 49 | Isobornyl acetate | C_12_H_20_O_2_ | 0.58±0.08^b^ | 0.84±0.02^a^ | - |
|  | 50 | Hexanedioic acid, bis(2-ethylhexyl) ester | C_22_H_42_O_4_ | - | 42.62±2.78^a^ | - |
|  | 51 | Silane glycol dimethyl ester | C_2_H_8_O_2_Si | - | 16.87±0.95^a^ | - |
|  | 52 | Propanoic acid, 2-hydroxy-, ethyl ester | C_5_H_10_O_3_ | - | 50.03±1.84^a^ | 52.03±7.1^a^ |
|  | 53 | Butanoic acid, 3-hydroxy-, ethyl ester | C_6_H_12_O_3_ | - | 1.72±0.34^b^ | 3.73±0.26^a^ |
|  | 54 | Pentanoic acid, 2-hydroxy-4-methyl-, ethyl ester | C_8_H_16_O_3_ | - | 8.57±0.54^a^ | 8.04±0.67^a^ |
|  | 55 | Benzeneacetic acid, ethyl ester | C_10_H_12_O_2_ | - | 1.42±0.02^b^ | 2.24±0.13^a^ |
|  | 56 | Nonanoic acid, ethyl ester | C_11_H_22_O_2_ | - | 3.66±0.04^a^ | - |
|  | 57 | Ethyl trans-4-decenoate | C_12_H_22_O_2_ | - | 2.45±0.22^a^ | - |
|  | 58 | 9-Octadecen-12-ynoic acid, methyl ester | C_19_H_32_O_2_ | - | 1.17±0.06^a^ | 1.08±0.066^a^ |
|  | 59 | Ethyl Acetate | C_4_H_8_O_2_ | - | 0.89±0.06^a^ | - |
|  | 60 | Butanoic acid, 2-methyl-, ethyl ester | C_7_H_14_O_2_ | - | - | 0.77±0.02^a^ |
|  | 61 | Hexanoic acid, 2-ethyl-, ethyl ester | C_10_H_20_O_2_ | - | - | 0.51±0.02^a^ |
|  | 62 | Acetic acid, nonyl ester | C_11_H_22_O_2_ | - | - | 0.49±0.01^a^ |
|  | 63 | Isosorbide Dinitrate | C_6_H_8_N_2_O_8_ | - | - | 0.5±0.04^a^ |
|  | 64 | Tetradecanoic acid, ethyl ester | C_16_H_32_O_2_ | - | - | 1.53±0.116^a^ |
|  | 65 | Hexyl n-valerate | C_11_H_22_O_2_ | - | - | 0.71±0.067^a^ |
| Ketones | 66 | 3-Heptanone, 2-methyl- | C_8_H_16_O | 2.53±0.3^a^ | - | - |
|  | 67 | 2-Nonanone | C_9_H_18_O | 4.63±0.15^a^ | 4.98±0.58^a^ | 4.96±0.06^a^ |
|  | 68 | 2-Cyclohexen-1-one, 3-methyl-6-(1-methylethyl)- | C_10_H_16_O | 4.27±0.37^a^ | 4.23±0.21^a^ | 3.73±0.36^b^ |
|  | 69 | 2-Undecanone | C_11_H_22_O | 4.26±0.4^a^ | 3.4±0.1^b^3 | 4.29±0.08^a^ |
|  | 70 | Xanthoxylin | C_10_H_12_O_4_ | 0.36±0.03^ab^ | 0.83±0.11^a^ | 0.48±0.04^ab^ |
| Acids | 71 | Acetic acid | C_2_H_4_O_2_ | 69.61±2.85^b^ | 74.92±3.15^a^ | 0.64±0.07^b^ |
|  | 72 | Hexanoic acid | C_6_H_12_O_2_ | 4.61±0.46 | 22.15±2.13^a^ | 19.5±1.53^ab^ |
|  | 73 | Nonanoic acid | C_9_H_18_O_2_ | 2.21±0.17^a^ | - | - |
|  | 74 | Acetic acid, 1,7,7-trimethyl-bicyclo[2.2.1]hept-2-yl ester | C_12_H_20_O_2_ | 1.3±0.03^a^ | - | 0.9±0.02^b^ |
|  | 75 | Butanoic acid | C_4_H_8_O_2_ | - | 13.15±0.72^a^ | 12.4±1.73^a^ |
|  | 76 | Pterin-6-carboxylic acid | C_7_H_5_N_5_O_3_ | - | 0.42±0.06^a^ | - |
|  | 77 | n-Decanoic acid | C_10_H_20_O_2_ | - | 3.02±0.08^b^ | 6.01±0.51^a^ |
|  | 78 | n-Hexadecanoic acid | C_16_H_32_O_2_ | - | 2.48±0.2^a^ | - |
|  | 79 | Ala-Gly | C_5_H_10_N_2_O_3_ | - | 0.73±0.06^a^ | - |
|  | 80 | Hexanoic acid, 2-methyl- | C_7_H_14_O_2_ | - | - | 1.51±0.05^a^ |
|  | 81 | Dodecanoic acid, 3-hydroxy- | C_12_H_24_O_3_ | - | - | 1.82±0.08^a^ |
| Olefins | 82 | 1,3,5,7-Cyclooctatetraene | C_8_H_8_ | 2.86±0.25^b^ | 6.2±0.19^a^ | 5.64±0.54^ab^ |
|  | 83 | β-Pinene | C_10_H_16_ | 7.33±0.51^a^ | - | - |
|  | 84 | Camphene | C_10_H_16_ | 2.62±0.38^b^ | - | 3.58±0.42^a^ |
|  | 85 | α-Phellandrene | C_10_H_16_ | 1.86±0.22^a^ | - | - |
|  | 86 | 1,3,6-Octatriene, 3,7-dimethyl-, (Z)- | C_10_H_16_ | 4.04±0.03^a^ | - | - |
|  | 87 | Caryophyllene | C_15_H_24_ | 4.92±0.01^ab^ | 4.9±0.12^ab^ | 5.41±0.2^a^ |
|  | 88 | Humulene | C_15_H_24_ | 0.9±0.09^b^ | 0.93±0.04^b^ | 1.86±0.11^a^ |
|  | 89 | Benzene, 1-(1,5-dimethyl-4-hexenyl)-4-methyl- | C_15_H_22_ | 10.4±0.27^a^ | 9.78±0.09^ab^ | 10.74±0.42^a^ |
|  | 90 | Cyclohexene, 3-(1,5-dimethyl-4-hexenyl)-6-methylene-, [S-(R*,S*)]- | C_15_H_24_ | 4.07±0.2^a^ | 4.51±0.87^a^ | 4.93±0.18^a^ |
|  | 91 | γ-Terpinene | C_10_H_16_ | - | 5.9±0.66^a^ | 5.75±0.03^a^ |
|  | 92 | 1-Octene, 3,7-dimethyl- | C_10_H_20_ | - | 9.23±0.37^a^ | - |
|  | 93 | Copaene | C_15_H_24_ | - | - | 0.98±0.01^a^ |
| Alkanes | 94 | Decane | C_10_H_22_ | 0.93±0.02^a^ | - | - |
|  | 95 | Tetradecane | C_14_H_30_ | 2.14±0.36^a^ | 2.07±0.07^a^ | 2.07±0.17^a^ |
|  | 96 | Undecane | C_11_H_24_ | - | 0.71±0.04^b^ | 1.83±0.03^a^ |
|  | 97 | Heptadecane, 2,6,10,14-tetramethyl- | C_21_H_44_ | - | - | 2.65±0.13^a^ |
| Phenols | 98 | Phenol | C_6_H_6_O | 9.33±0.19^a^ | 3.21±0.3^b^ | 3.68±0.09^b^ |
|  | 99 | Eugenol | C_10_H_12_O_2_ | - | - | 2.11±0.08^a^ |
| Benzene | 100 | P-Xylene | C_8_H_10_ | 3.55±0.37^b^ | 5.32±0.63^a^ | 2.9±0.04^bc^ |
|  | 101 | Benzene, 1-ethyl-3-methyl- | C_9_H_12_ | 4.1±0.74^b^ | - | 5.23±0.2^a^ |
|  | 102 | Benzene, 1-methyl-2-(1-methylethyl)- | C_10_H_14_ | 8.91±0.03^a^ | - |  |
|  | 103 | Ethylbenzene | C_8_H_10_ | - | 0.74±0.03^ab^ | 1.07±0.02^a^ |
|  | 104 | Benzene, 1,2,4-trimethyl- | C_9_H_12_ | - | - | 0.71±0.04^a^ |

Data are presented as mean ± standard deviation. Different letters at the same row indicate significant difference (*p* < 0.05). - indicate not checked out.
