# Supplementary material for: Effect of Limosilactobacillus fermentum 332 on physicochemical characteristics, volatile flavor components, and Quorum sensing in fermented sausage
Source: Sci Rep. 2023 Mar 9;13:3942. doi: 10.1038/s41598-023-31161-2 (PMC9998864; doi:10.1038/s41598-023-31161-2)
Supplement: Supplementary file 2 — Supplementary Table S2. [file 41598_2023_31161_MOESM2_ESM.docx]

**Table S2** The volatile flavor compounds of fermented sausages in the control

| Volatile compounds |  | Name | Molecular formula | 1 d | 5 d | 11 d |
| --- | --- | --- | --- | --- | --- | --- |
| Alcohols | 1 | Ethanol | C_2_H_6_O | 59.4±1.35^a^ | 0.55±0.006^b^ | - |
|  | 2 | 2,3-Butanediol | C_4_H_10_O_2_ | 30.61±0.28^a^ | - | - |
|  | 3 | 2-Hexanol, 3-methyl- | C_7_H_16_O | 1.44±0.03 ^a^ | 1.4±0.01 ^a^ | - |
|  | 4 | 1-Heptanol, 6-methyl- | C_8_H_18_O | 5.19±0.12 ^a^ | - | - |
|  | 5 | 1,6-Octadien-3-ol,3,7-dimethyl- | C_10_H_18_O | 31.55±1.75^b^ | 33.62±4.8^a^ | 32.46±0.29^a^ |
|  | 6 | endo-Borneol | C_10_H_18_O | 10.41±0.32^a^ | - | 11.15±0.53^a^ |
|  | 7 | 2-Octen-1-ol, 3,7-dimethyl- | C_10_H_20_O | 1.34±0.1^a^ | - | - |
|  | 8 | Terpinen-4-ol | C_10_H_18_O | 4.5±0.21^a^ | 4.75±0.89^a^ | 4.42±0.39^a^ |
|  | 9 | 2,3-Butanediol, [S-(R*,R*)]- | C_4_H_10_O_2_ | 36.07±1.02^b^ | - | 42.41±3.07^a^ |
|  | 10 | 1-Butanol, 3-methyl- | C_5_H_12_O | 12.71±0.1^a^ | - | - |
|  | 11 | α-Terpineol | C_10_H_18_O | 20.72±0.2^b^ | - | 26.76±0.66^a^ |
|  | 12 | Eucalyptol | C_10_H_18_O | 143.46±6.09^a^ | 128.98±6.88^b^ | 111.86±7.45^c^ |
|  | 13 | 1-Octen-3-ol | C_8_H_16_O | 3.65±0.05^a^ |  | 3.49±0.05^a^ |
|  | 14 | 2,6-Octadien-1-ol, 3,7-dimethyl-, (Z)- | C_10_H_18_O | - | 4.81±0.79^a^ | 4.43±0.29^a^ |
|  | 15 | Diethylene glycol | C_4_H_10_O_3_ | - | 14.93±0.89^a^ | 7.01±0.11^b^ |
|  | 16 | 2,3-Butanediol, [R-(R*,R*)]- | C_4_H_10_O_2_ | - | 32±1.93^a^ | 25.44±3.09^b^ |
|  | 17 | 1-Octanol | C_8_H_18_O | - | 9.13±0.31^ab^ | 10.87±0.25^a^ |
|  | 18 | 2-Hexadecanol | C_16_H_34_O | - | 1.19±0.09^a^ | 0.43±0.07^b^ |
|  | 19 | 1-Dodecanol | C_12_H_26_O | - | 3.81±0.09^a^ | - |
| Aldehyde | 20 | Heptanal | C_7_H_14_O | 6.53±0.68^b^ | 38.49±7.159^a^ | 35.68±1.01^a^ |
|  | 21 | Nonanal | C_9_H_18_O | 37.07±2.67^b^ | 142.89±20.36^a^ | 146.72±2.46^a^ |
|  | 22 | Decanal | C_10_H_20_O | 2.56±0.42^b^ | 4.35±0.42^ab^ | 5.5±0.38^a^ |
|  | 23 | 2-Nonenal, (E)- | C_9_H_16_O | 0.65±0.06^b^ | 1.38±0.08^a^ | 1.43±0.09^a^ |
|  | 24 | Octanal | C_8_H_16_O | - | 37.2±5.81^a^ | 37.53±0.47^a^ |
|  | 25 | Succindialdehyde | C_4_H_6_O_2_ | - | 0.24±0.1^a^ | - |
|  | 26 | Undecanal | C_11_H_22_O | - | - | 0.66±0.01^a^ |
|  | 27 | Benzaldehyde | C_7_H_6_O | - | - | 5.23±1.13^a^ |
| Esters | 28 | Ethyl Acetate | C_4_H_8_O_2_ | 5.16±0.06^a^ | - | - |
|  | 29 | Butanoic acid, ethyl ester | C_6_H_12_O_2_ | 17.38±1.48^b^ | 27.31±1.12^a^ | 29.67±5.58^a^ |
|  | 30 | Ethyl caproate | C_8_H_16_O_2_ | 74.48±0.19^c^ | 126.81±9.64^a^ | 118.22±7.32^b^ |
|  | 31 | 3-Cyclohexene-1-methanol, α,α,4-trimethyl-, acetate | C_12_H_20_O_2_ | 4.88±0.1^c^ | 5.45±0.33^b^ | 7.71±1.28^a^ |
|  | 32 | Ethyl octanoate | C_10_H_20_O_2_ | 32.53±5.94^c^ | 63.57±5.96^b^ | 75.76±0.94^a^ |
|  | 33 | 1,6-Octadien-3-ol, 3,7-dimethyl-, 2-aminobenzoate | C_17_H_23_NO_2_ | 3.13±0.23^b^ | 5.22±0.92^a^ | 5.58±0.22^a^ |
|  | 34 | Nonanoic acid, ethyl ester | C_11_H_22_O_2_ | 1.58±0.22^b^ | - | 4.22±0.09^a^ |
|  | 35 | Decanoic acid, ethyl ester | C_12_H_24_O_2_ | 9.89±1.48^c^ | 30.89±5.45^b^ | 40.25±1.17^a^ |
|  | 36 | 5,8,11,14-Eicosatetraenoic acid, methyl ester, (all-Z)- | C_21_H_34_O_2_ | 0.54±0.01^a^ | - | 0.55±0.03^a^ |
|  | 37 | Dodecanoic acid, ethyl ester | C_14_H_28_O_2_ | 0.75±0.11^c^ | 2.87±0.27^ab^ | 3.02±0.44^a^ |
|  | 38 | Hexadecanoic acid, ethyl ester | C_18_H_36_O_2_ | 0.38±0.02^b^ | 1.1±0.12^a^ | 1.13±0.05^a^ |
|  | 39 | Butanoic acid, 3-methyl-, ethyl ester | C_7_H_14_O_2_ | 7.8±0.5^b^ | 12.87±0.19^a^ | 11.17±1.65^a^ |
|  | 40 | Allyl 2-ethyl butyrate | C_9_H_16_O_2_ | 0.19±0.01^a^ | - | - |
|  | 41 | Nerolidyl acetate | C_17_H_28_O_2_ | 0.45±0.03^a^ | - | - |
|  | 42 | Pentanoic acid, ethyl ester | C_7_H_14_O_2_ | 1.23±0.14^b^ | 2.45±0.17^a^ | 2.39±0.26^a^ |
|  | 43 | Isobornyl acetate | C_12_H_20_O_2_ | 0.9±0.08^a^ | - | 0.86±0.1^a^ |
|  | 44 | 1-Butanol, 3-methyl-, formate | C_6_H_12_O_2_ | 6.09±1.66^a^ | - | 4.64±0.55^b^ |
|  | 45 | Propanoic acid, 2-hydroxy-, ethyl ester | C_5_H_10_O_3_ | - | 17.48±2.51^a^ | - |
|  | 46 | Ethyl trans-4-decenoate | C_12_H_22_O_2_ | - | 1.89±0.2^b^ | 2.21±0.1^a^ |
|  | 47 | Isosorbide Dinitrate | C_6_H_8_N_2_O_8_ | - | 1.77±0.46^a^ | 1.16±0.49^b^ |
|  | 48 | Nonanoic acid, ethyl ester | C_11_H_22_O_2_ | - | 3.58±0.58^a^ | - |
|  | 49 | 9-Octadecen-12-ynoic acid, methyl ester | C_19_H_32_O_2_ | - | 1.07±0.13^a^ | 1.22±0.01^a^ |
|  | 50 | Propanoic acid, 2-hydroxy-, ethyl ester, (S)- | C_5_H_10_O_3_ | - | - | 24.72±10.21^a^ |
|  | 51 | Butanoic acid, 3-hydroxy-, ethyl ester | C_6_H_12_O_3_ | - | - | 2.22±0.01^a^ |
|  | 52 | Hexyl n-valerate | C_11_H_22_O_2_ | - | - | 0.56±0.02^a^ |
|  | 53 | Geranyl isovalerate | C_15_H_26_O_2_ | - | - | 0.7±0.06^a^ |
|  | 54 | Tetradecanoic acid, ethyl ester | C_16_H_32_O_2_ | - | - | 1.35±0.05^a^ |
|  | 55 | 1,5-Dimethyl-1-vinyl-4-hexenyl butyrate | C_14_H_24_O_2_ | - | - | 0.91±0.01^a^ |
| Ketones | 56 | 3-Heptanone, 2-methyl- | C_8_H_16_O | 5.23±0.68^a^ | - | - |
|  | 57 | 2-Nonanone | C_9_H_18_O | 3.58±0.04^a^ | 3.77±0.526^a^ | 3.5±0.07^a^ |
|  | 58 | 2-Cyclohexen-1-one, 3-methyl-6-(1-methylethyl)- | C_10_H_16_O | 3.05±0.34^b^ | 4.03±0.74^a^ | 4.21±0.35^a^ |
|  | 59 | 2-Undecanone | C_11_H_22_O | 2.03±0.27^b^ | 4.17±0.44^a^ | 4.58±0.38^a^ |
|  | 60 | 2-Butanone, 3-hydroxy- | C_4_H_8_O_2_ | 13.49±1.62^a^ | - | - |
|  | 61 | Xanthoxylin | C_10_H_12_O_4_ | - | 0.61±0.07^a^ | 0.51±0.11^a^ |
| Acids | 62 | Acetic acid | C_2_H_4_O_2_ | 22.32±2.43^b^ | 3.04±0.22^c^ | 51.22±0.97^a^ |
|  | 63 | Butanoic acid | C_4_H_8_O_2_ | 4.29±0.3^b^ | 14.42±2.9^a^ | 15.01±3.5^a^ |
|  | 64 | Hexanoic acid | C_6_H_12_O_2_ | 2.23±0.16^c^ | 23.86±4.33^b^ | 29.13±1.57^a^ |
|  | 65 | Hexanoic acid, 2-ethyl- | C_8_H_16_O_2_ | 0.52±0.01^a^ | - | - |
|  | 66 | Oleic Acid | C_18_H_34_O_2_ | 0.36±0.02^a^ | - | - |
|  | 67 | Butanoic acid, 2-methyl- | C_5_H_10_O_2_ | - | 2.22±0.46^a^ | - |
|  | 68 | Dodecanoic acid, 3-hydroxy- | C_12_H_24_O_3_ | - | 0.64±0.08^a^ | 0.51±0.02^a^ |
|  | 69 | Pterin-6-carboxylic acid | C_7_H_5_N_5_O_3_ | - | 0.51±0.02^a^ | - |
|  | 70 | n-Decanoic acid | C_10_H_20_O_2_ | - | 6.55±0.97^a^ | 4.64±0.38^b^ |
|  | 71 | Hexanoic acid, 2-methyl- | C_7_H_14_O_2_ | - | - | 2.54±0.12^a^ |
| Olefins | 72 | 1,3,5,7-Cyclooctatetraene | C_8_H_8_ | 2.26±0.2^b^ | 3.48±0.26^a^ | 3.94±0.64^a^ |
|  | 73 | α-Pinene | C_10_H_16_ | 5.05±0.34^a^ | - | - |
|  | 74 | Camphene | C_10_H_16_ | 2.61±0.15^a^ | - | 2.7±0.01^a^ |
|  | 75 | β-Pinene | C_10_H_16_ | 6.92±0.25^a^ | - | - |
|  | 76 | α-Phellandrene | C_10_H_16_ | 3.03±0.08^a^ | - | - |
|  | 77 | Cyclohexene, 3-(1,5-dimethyl-4-hexenyl)-6-methylene-, [S-(R*,S*)]- | C_15_H_24_ | 2.24±0.06^b^ | - | 5.49±0.45^a^ |
|  | 78 | γ-Terpinene | C_10_H_16_ | 2.95±0.37^b^ | 2.94±0.55^b^ | 4.26±0.1^a^ |
|  | 79 | Caryophyllene | C_15_H_24_ | 3.67±0.89^b^ | 5.27±0.56^a^ | 5.49±0.25^a^ |
|  | 80 | Copaene | C_15_H_24_ | 0.65±0.03^a^ | - | - |
|  | 81 | Humulene | C_15_H_24_ | 0.55±0.08^c^ | 1.84±0.43^b^ | 3±0.7^a^ |
|  | 82 | Benzene, 1-(1,5-dimethyl-4-hexenyl)-4-methyl- | C_15_H_22_ | 7.31±0.37^b^ | 11.46±1.75^a^ | - |
|  | 83 | (1S)-2,6,6-Trimethylbicyclo[3.1.1]hept-2-ene | C_10_H_16_ | - | 4.45±0.21^a^ | - |
|  | 84 | 1,3,6-Octatriene, 3,7-dimethyl-, (Z)- | C_10_H_16_ | - | - | 4.58±0.06^a^ |
| Alkanes | 85 | Decane | C_10_H_22_ | 0.77±0.13^a^ | - | - |
|  | 86 | Tetradecane | C_14_H_30_ | 0.88±0.05^b^ | 2.85±0.32^a^ | 2.54±0.63^a^ |
|  | 87 | Undecane | C_11_H_24_ | 1.43±0.14^a^ | 1.77±0.056^a^ | 1.74±0.17^a^ |
|  | 88 | Heptadecane, 2,6,10,14-tetramethyl- | C_21_H_44_ | - | 2.81±0.34^a^ | 2.85±0.17^a^ |
| Phenols | 89 | Phenol | C_6_H_6O_ | - | - | 6.56±0.42^a^ |
| Benzene | 90 | P-Xylene | C_8_H_10_ | 3.6±0.32^b^ | - | 5.34±0.03^a^ |
|  | 91 | Benzene, 1-ethyl-3-methyl- | C_9_H_12_ | 0.24±0.02^c^ | 5.71±0.053^a^ | 4.65±0.1^b^ |
|  | 92 | Benzene, 1-methyl-2-(1-methylethyl)- | C_10_H_14_ | 8±0.43^a^ | - | - |
|  | 93 | Mesitylene | C_9_H_12_ | - | 0.81±0.03^a^ | - |
|  | 94 | Benzene, 1,2,4-trimethyl- | C_9_H_12_ | - | - | 0.73±0.03^a^ |
|  | 95 | Ethylbenzene | C_8_H_10_ | - | - | 0.75±0.06^a^ |

Data are presented as mean ± standard deviation. Different letters at the same row indicate significant difference (*p* < 0.05). - indicate not checked out.
